# An Aggressive Case of Cryoglobulinemia and Membranoproliferative Glomerulonephritis: A Case Report

**DOI:** 10.7759/cureus.62193

**Published:** 2024-06-11

**Authors:** Leonard Ferdman, Hannah Jensen, Alshaimaa Hazaa, Robert W Donnell

**Affiliations:** 1 Internal Medicine, University of Arkansas for Medical Sciences, Fayetteville, USA; 2 Department of Surgery, University of Arkansas for Medical Sciences, Fayetteville, USA; 3 Department of Internal Medicine, Mercy Hospital, Rogers, USA

**Keywords:** autoimmune vasculitis, rheumatology & autoimmune diseases, hepatitis c (hcv) infection, membranoproliferative glomerulonephritis (mpgn), cryoglobulinemia vasculitis

## Abstract

This case report describes a 66-year-old female with membranoproliferative glomerulonephritis (MPGN) with pulmonary involvement presumed secondary to Hepatitis C virus (HCV)-associated with mixed cryoglobulinemia. In this condition, pulmonary involvement is uncommon, and aggressive lung involvement can be associated with poor outcomes. Within eight weeks, the patient was hospitalized twice with acute pulmonary presentations and presented at a third hospitalization with dyspnea, chest pain, abdominal pain, and edema. Imaging revealed persistent and historically evolving lung consolidation, as well as a renal biopsy showing MPGN associated with mixed cryoglobulinemia. A lung biopsy revealed inflammation. Bronchoalveolar lavage did not show hemosiderin-laden macrophages and did not grow infectious agents. Serology revealed negative ANCAs and rheumatoid factor positive at 476 IU/ml (upper limit normal 14 IU/ml). Qualitative cryoglobulins were positive at 2 %ppt (reference range: negative %ppt) and Type II mixed cryoglobulinemia with IgM kappa plus polyclonal IgG. The treatment involved steroids and rituximab. The patient’s clinical status deteriorated, and she elected to change her resuscitation status to comfort care measures. This case emphasizes that cryoglobulinemia can present with aggressive manifestations on a wide spectrum. Pulmonary manifestations are rare and were evident in this case (although without clear evidence of diffuse alveolar hemorrhage) and led to a complicated disease course and an unfavorable outcome. Overall, this case underscores the complexity of mixed cryoglobulinemia presentations and the challenges of managing severe cases with multi-organ involvement.

## Introduction

Chronic hepatitis C infection (HCV) can lead to stimulation of the immune system and the development of cryoglobulinemia [1-4]. Cryoglobulinemia is defined as the active presence of serum cryoglobulins, or immunoglobulins, that reversibly precipitate below 37°C. It can often occur in an asymptomatic fashion, although palpable purpura is the most commonly reported symptom [3]. Cryoglobulinemia is further categorized into Type I, Type II, and Type III. Type I includes a purely monoclonal immunoglobulin presence. Type II (T2MC) is considered “mixed” and occurs as monoclonal rheumatoid factor (RF) IgM and polyclonal IgG [3]. Type III cryoglobulinemia is also considered "mixed,” as it includes polyclonal IgM and polyclonal IgG [3]. HCV is understood to be associated with the development of T2MC [3]. While the full spectrum of this immunological mechanism of action is not fully understood, it is known that HCV-induced cryoglobulinemia involves the hyperactivity of B-cells [3]. We present a case of T2MC and associated membranoproliferative glomerulonephritis (MPGN) with uniquely aggressive renal and pulmonary pathology in an HCV-positive patient.

## Case presentation

A 66-year-old female with chronic obstructive pulmonary disease (COPD), congestive heart failure, gastroesophageal reflux disease, asthma, and pulmonary hypertension was hospitalized for COPD exacerbation and spontaneous right pneumothorax. Three weeks after discharge, she was then again admitted, treated for a suspected lung abscess, and discharged with intravenous metronidazole and ceftazidime. During those hospitalizations, her microbiological studies did not isolate any infectious organisms. A week later, she decompensated and was again hospitalized for a third time for dyspnea, chest pain, abdominal pain, abdominal swelling, bilateral lower extremity swelling, and decreased urine output. A physical examination reviewed abdominal tenderness and distention, wheezes throughout the lung fields, edema in the lower extremities, and very minimal urine production. Creatinine was elevated at 1.48 mg/dL (reference range 0.6-1.1 mg/dL), with a baseline range of around 0.6 mg/dL. Urinalysis showed 3+ proteinuria, 3+ blood, 51-100 RBC/hpf, and 3-5 WBC/hpf.

**Figure 1:**
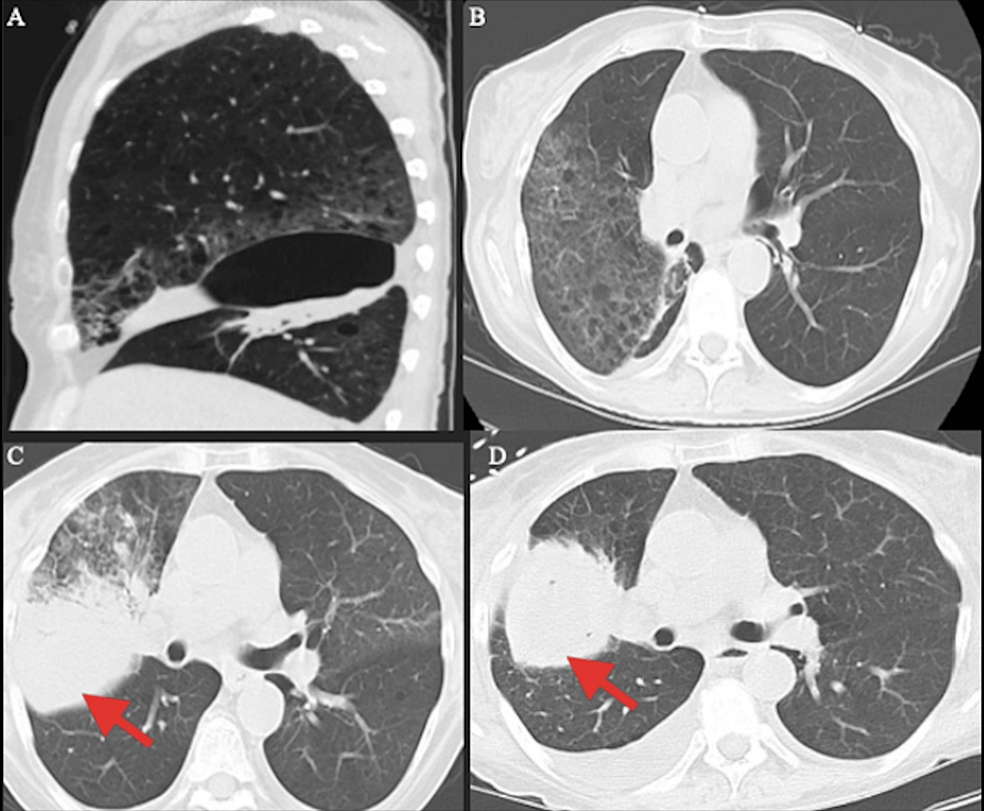
Computed tomography imaging findings over three hospital admissions A) First admission: complete consolidation of the right middle lobe. Right upper lobe (RUL) with a ground glass appearance. Large oval air bulla along the right major fissure. B) First admission: ground glass appearances. C) Second admission: increased consolidative changes in the RUL and small lung pleural effusion. D) Third admission: mass-like consolidation in RUL and increased size of right-sided effusion.

Computed tomography (CT) and chest X-rays showed the persistence of a mass-like consolidation in the right upper lung lobe described during her prior hospitalization. Additional imaging findings included pleural effusions and pulmonary edema. CT imaging findings over three hospital admissions can be seen in Figure 1 (mass-like consolidation and evolution can be visualized by red arrows). Initially, our patient was treated with antibiotics and aggressive diuresis. Given both pulmonary and renal findings, a workup for pulmonary renal syndrome (PRS) was initiated. A CT-guided lung biopsy showed inflammation and fibrosis, findings consistent with organizing pneumonia. The lung biopsy also showed hemosiderin deposition throughout. Diffuse alveolar hemorrhage (DAH) was not observed on imaging, and bronchioalveolar lavage (BAL) did not demonstrate hemosiderin-laden macrophages (HLM). A renal biopsy showed MPGN, and hyaline deposits associated with mixed cryoglobulinemia. The patient was found to be RF positive with a titer of 476 and decreased C3 and C4 levels. Qualitative cryoglobulins were positive at 2 %ppt (reference range: negative %ppt) and determined to be T2MC with IgM kappa plus polyclonal IgG. She was found to be HCV-positive with an active viral load. All other antibody screens were found to be negative, including ANCA. The patient was treated with steroids and rituximab. During her hospitalization, she at one point required intubation and placement in the intensive care unit. When she returned to the medical floor, she continued to experience dyspnea, malaise, and anxiety. Due to the severity of recurrent episodes of dyspnea and lethargy, the patient elected to change her resuscitation status to comfort care measures. Her diagnoses were MPGN, T2MC, and organizing pneumonia.

## Discussion

The prevalence of mixed cryoglobulins in patients with chronic HCV infection is reported to be about 40-50%, and the development of symptomatic cryoglobulin-associated vasculitis in chronic HCV-infected individuals is believed to be 5% [4,5]. The common clinical manifestations of T2MC are purpura and peripheral neuropathy [4]. A more aggressive manifestation of T2MC in the setting of HCV infection is renal pathology secondary to MPGN [4]. In the context of the development of T2MC glomerular disease, MPGN appears to occur most frequently [6]. The survival of T2MC begins to diminish when renal disease develops. One study found that in a group of 119 T2MC cases in HCV+ patients, the five-year survival rate (in a group of 103 patients) without renal disease was 87%, while the survival rate (in a group of 16 patients) was 50% [7]. Another study that looked at 29 cases of life-threatening cryoglobulinemia found that those with pulmonary hemorrhage and intestinal ischemia had 100% mortality [8]. While pulmonary manifestations are not typical, lung involvement in T2MC can result in a much more complicated disease process, as evident in our patient’s case. In essence, the current literature has shown that pulmonary involvement in T2MC presenting with alveolar hemorrhage is a poor prognostic factor [8-11]. There have been several case reports that have cited pulmonary manifestations of T2MC [9, 12-15]. Our patient did not have DAH shown on imaging. Additionally, BAL did not demonstrate HLM based on the results. However, lung biopsy results did indicate hemosiderin deposition, which does point towards evidence of potential alveolar hemorrhaging that may have been too mild to visualize on imaging or still in the early stages. As to whether alveolar hemorrhaging was present (and its extent) or not, it was simply not determinable. Figure 1 demonstrates radiological lung field findings during three recurrent hospitalizations. Initially, the evolving infiltrates appeared to be infectious, but despite broad-spectrum antibiotic coverage, they did not resolve. As there appeared to be a persistence of right-sided lung consolidation, one can appreciate the degree of involvement that T2MC can have. It can also be observed that such findings are similar to and nearly indiscernible from pneumonia and, in our patient’s case, likely recurrent pneumonia from her prior admission. It should also be noted that despite purpura being a very common manifestation of cryoglobulinemia, our patient did not experience this finding despite the severity of her disease burden.﻿

## Conclusions

This case report demonstrated a unique case of T2MC with pulmonary involvement in the form of persistent mass-like consolidation and organizing pneumonia that required mechanical ventilation in an intensive care unit. This case report aims to highlight and reiterate the importance of recognizing the seriousness of pulmonary manifestations in T2MC. This case report also highlights the importance of HCV screening for those who are age-appropriate and high-risk, that is, all individuals who are 18 years of age or older. High-risk individuals for HCV infection include but are not limited to, those with employment involving needles, IV drug use, babies born to HCV-infected mothers, and high-risk sexual behavior. Future studies should continue to investigate the pathogenesis of HCV-associated T2MC and how this disease affects visceral organs. Future research should also focus on further understanding the mechanism of action and management of pulmonary involvement in cryoglobulinemia to improve outcomes in such individuals.
